# Efficient extraction of bioactive flavonoids from *Celtis sinensis* leaves using deep eutectic solvent as green media†

**DOI:** 10.1039/d1ra01848e

**Published:** 2021-05-18

**Authors:** Lei Wang, Xianying Fang, Yang Hu, Yiwei Zhang, Zhipeng Qi, Jie Li, Linguo Zhao

**Affiliations:** College of Chemical Engineering, Nanjing Forestry University Nanjing 210037 China njfu2304@163.com +86-25-85427396; Co-Innovation Center for Sustainable Forestry in Southern China, Nanjing Forestry University Nanjing 210037 China; Jiangsu Co-Innovation Center of Efficient Processing and Utilization of Forest Resources, Nanjing Forestry University Nanjing 210037 China

## Abstract

In recent years, deep eutectic solvent (DES) has attracted comprehensive attention on the extraction of natural products, and is regarded as an alternative to traditional organic solvents for the environmental advantages. Twenty-six DESs were compared for their extraction yield of total flavonoids and isovitexin (the main flavonoid in *Celtis sinensis*) from *Celtis sinensis*. The results show that the extraction yields of total flavonoids by betaine/glycolic acid (DES8), ethylamine hydrochloride/1,2-propanediol (DES12) and tetrapropylammonium bromide/lactic acid (DES17) are the highest, while the extraction yields of isovitexin by ethylene glycol/malonic acid (DES23), ethylene glycol/glycolic acid (DES24) and 1,2-propanediol/glycolic acid (DES26) are the highest. The extraction conditions using the above six DESs were further optimized systematically. Under optimum conditions, the extraction rates of total flavonoids and isovitexin can be increased up to 95.39 and 10.58 mg g^−1^, respectively, which were significantly higher than that of methanol extraction. In order to exclude the effect of DESs on the bioactivity of *Celtis sinensis* extract, the macroporous resin D-101 was used to purify the total flavonoids from DESs extract, and the recovery rates of flavonoids from the above six kinds of DESs were all over 80%. Next, the anti-inflammatory activity of DES extracts was compared using a lymphocyte transformation experiment. The result showed that the inhibition rate of the DES24 extract on the proliferation of Con A-activated T cells was up to 72% with an IC_50_ value of 124.8 μg mL^−1^. None of the DESs extracted exhibited cytotoxicity on normal T cells. The mechanism of the anti-inflammatory activity against Con A-activated T cells may be that DES24 flavonoids extract induced the apoptosis of inflammatory T cells, and activated the expression of pro-apoptotic protein. Taken together, DES has showed significant advantages on the extraction of natural products for the relatively mild extraction condition, high yield and environmental-friendliness.

## Abstract 2

In recent years, deep eutectic solvent (DES) has attracted comprehensive attention on the extraction of natural products, and is regarded as an alternative to traditional organic solvents for the environmental advantages.

## Graphical abstract

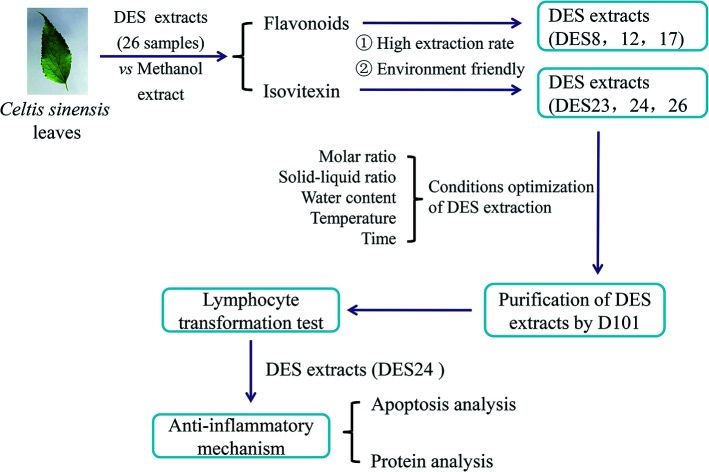

## 1. Introduction

*Celtis sinensis* Pers. is a kind of deciduous tree that belongs to the ulmus family, and mostly grows in plain and shade-tolerant areas. *Celtis* has been widely used as a folk medicine to treat stomach diseases, lumbago, abdominal pain, urticaria, eczema and other diseases in Korea, Japan and China. At present, a variety of properties, such as antioxidant, anti-tumor, antibacterial, and anti-inflammatory, inhibit acetylcholinesterase (AChE), and other activities of chemical components have been found from *Celtis* by international scholars. At the same time, the pharmacology and toxicology of flavonoids and terpenoids in *Celtis sinensis* have been further studied.^1^

There are many components in *Celtis sinensis*, such as anthraquinones, phenols and triterpenes. The content of flavonoids in *Celtis sinensis* is abundant and varied, such as quercetin, rutin, isovitexin, and cytisoside. These compounds have medicinal properties, and few studies on the extraction and separation of *Celtis sinensis* have been reported. There are reports suggesting the chemical constituents and pharmacological activities of different parts in *Celtis sinensis*. The bark, fruit and fatty acids in the fruit of the *Celtis australis* were used as the research objects to investigate the anti-inflammatory activity by selecting rat foot swelling as a model. The cytotoxicity and anti-tumor activity of *Celtis australis* and *Celtis occidentalis* have been tested.^2^ Other studies have shown that the *Celtis sinensis* leaves have a certain analgesic effect and anti-mutation activity,^3^ and they have also shown a protective effect on gastric mucosa.^4^ At present, traditional organic solvents are still used to extract the relevant chemical components. *Celtis australis* and *Celtis occidentalis* were taken as the research objects to extract the effective components, and eight monomer compounds were isolated by researchers.^2^

It is well known that the flammability and volatility of traditional organic solvents cause environmental pollution. In recent years, people have paid more and more attention to green technology. Green solvent is one of the important contents of green technology. In 2003, Abbott first prepared the solution formed by the mixture of choline chloride and urea. Thus, the concept of DES (deep eutectic solution) was proposed.^5^ DES has been considered as a green solvent because of its environment-friendliness, and as a safe alternative to the conventional organic solvents.^6^ In fact, DES has been used in the fields of catalysis, organic synthesis, dissolution, electrochemistry and material chemistry to improve the efficiency and reduce pollution.^7,8^ DES is a low eutectic mixture composed of a hydrogen bond acceptor (HBA) and hydrogen bond donor (HBD) with a certain molar ratio.^9^ DES has a broad application prospect as a solvent for use in many fields because of its biodegradability, low toxicity, easy preparation, and novel properties.^9,10^ There have been many reports on the extraction of bioactive compounds from plants by using hydrophilic DES as the extraction media. DES has also been reported in the extraction of phenolic compounds^11,12^ anthocyanin,^13^ ginsenoside,^14^ catechin,^15^ flavonoids,^15–19^ and more applications are still being explored.

There have been no studies on the application of DES to extract the total flavonoids and isovitexin from *Celtis sinensis* leaves. Consequently, this work looks at the possibility of DES increasing the concentration of extracted flavonoids and isovitexin compared with methanol extraction. The effect of DES conditions (hydrogen bond receptors, HBA/HBD ratio and water content) and extraction conditions (temperature, DESs/solid ratio and time) on the extraction efficiency were investigated systematically.

It has been reported that ginkgolic acid (GA) may cause allergic contact dermatitis (ACD) when people wash the pericarp of the rotting ginkgo apricot.^11,20^ The reason for people getting ACD is GA, as a hepten, with the ability to activate T lymphocytes against innocuous or auto-antigens and induce type-IV allergic reactions.^21^ The leaves of *Celtis* are employed as a folk medicine for dermatitis diseases caused by urushiol.^22^ Urushiol, one of the most commonly encountered contact allergens, has a chemical structure similar to ginkgolic acid, which causes ACD. With these claims, the effect of extracts purified with macroporous resin on the cytotoxicity of Con A-induced T lymphocyte was explored.

## 2. Materials and methods

### 2.1. Sample collection and materials

The raw material of the experiment was *Celtis sinensis* leaves (Fig. S1†) obtained from the Guizhou province in October 2018. The leaves were dried at 60 °C until a constant weight was achieved, and then pulverized to 30–40 meshes by a disintegrator. The pulverized material was stored in desiccators prior to use.

All compounds of analytical reagent grade used for DESs preparation were purchased from Aladdin Chemistry Co., Ltd. (Shanghai, China), and used without further purification. HPLC-grade methanol was obtained from Tedia Company., Inc. (Shanghai, China). Deionized water was obtained by a Milli-Q water purification system (Millipore, Billerica, MA). The rutin and isovitexin were purchased from Sigma-Aldrich (St. Louis, MO, USA), and their purities were more than 98%.

The macroporous resin (D-101) used to recover the flavonoids from the DES extraction solutions were bought from Changzhou Bon Adsorber Technology Co., Ltd. (Changzhou, China).

### 2.2. Preparation of deep eutectic solvents

DESs were prepared by mixing a hydrogen bond acceptor with a donor in proportions, as listed in Table 1. In addition to using choline chloride, betaine, ethylamine hydrochloride and tetrapropylammonium bromide as HBA and alcohols or acids as HBD to prepare DES, we tried to use alcohols, such as ethylene glycol, 1,2-propylene glycol as one component, and some acids as another component to prepare DES. DES was labeled as component 1 and component 2.^23^ DES was prepared by heating different component mixtures to 90 °C under a certain molar ratio, and constantly stirring until a homogeneous liquid was formed, and constantly stirring until a clear solution was obtained.^24^ The 26 kinds of prepared DES were stored at room temperature.

**Table 1:** The types of deep eutectic solvent used in this work

Abbreviation	Component 1	Component 2	Molar ratio
DES1	Choline chloride	Glycerin	1 : 2
DES2	Choline chloride	Urea	1 : 2
DES3	Choline chloride	Lactic acid	1 : 2
DES4	Choline chloride	Ethylene glycol	1 : 2
DES5	Choline chloride	Malonic acid	1 : 2
DES6	Choline chloride	1,2-Propanediol	1 : 2
DES7	Choline chloride	Glycolic acid	1 : 2
DES8	Betaine	Glycolic acid	1 : 2
DES9	Betaine	Lactic acid	1 : 2
DES10	Betaine	Glycerin	1 : 2
DES11	Ethylamine hydrochloride	Ethylene glycol	1 : 2
DES12	Ethylamine hydrochloride	1,2-Propylene glycol	1 : 2
DES13	Ethylamine hydrochloride	Lactic acid	1 : 2
DES14	Ethylamine hydrochloride	Glycerin	1 : 2
DES15	Ethylamine hydrochloride	Glycolic acid	1 : 2
DES16	Tetrapylammonium bromide	1,2-Propylene glycol	1 : 2
DES17	Tetrapylammonium bromide	Lactic acid	1 : 2
DES18	Tetrapylammonium bromide	Glycerin	1 : 2
DES19	Tetrapylammonium bromide	Glycolic acid	1 : 2
DES20	Tetrapylammonium bromide	Malonic acid	1 : 2
DES21	Ethylene glycol	Lactic acid	1 : 2
DES22	Ethylene glycol	Malic acid	1 : 2
DES23	Ethylene glycol	Malonic acid	1 : 2
DES24	Ethylene glycol	Glycolic acid	1 : 2
DES25	1,2-Propylene glycol	Malonic acid	1 : 2
DES26	1,2-Propylene glycol	Glycolic acid	1 : 2

### 2.3. Extraction of total flavonoids and isovitexin from *Celtis sinensis*

For the initial DES screening, an accurately weighed 100 mg sample of *Celtis sinensis* leaves powder was mixed with 1.0 mL of extraction solvent (water-added DES) in a 10.0 mL Eppendorf tube. After brief vortexing, the mixture was extracted in the shaking water bath with an extracting condition at 200 rpm and 40 °C for 10 min, and then centrifuged at 8000 × *g* for 20 min. The suspensions were filtered through a 0.45 μm membrane prior to HPLC analysis for detecting the content of isovitexin and rutin test for the total flavonoids. The repeatability was tested by extracting the *Celtis* powders three times over to evaluate the samples.

### 2.4. Determination of bioactive compounds in *Celtis sinensis* leaves

**Fig. 1:**
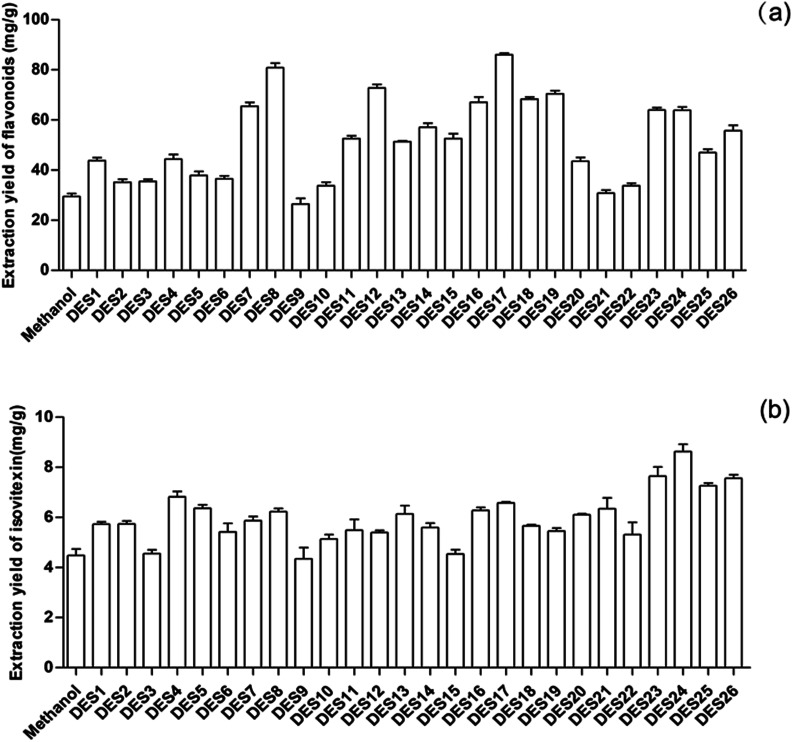
Extraction yields of the total flavonoids and isovitexin from *Celtis sinensis* leaves by different extraction solvents. Twenty-six kinds of DESs containing 30% (w/w) water were used as extraction solvents at a molar ratio of 1 : 2 and a solvent-to-solid ratio of 20 : 1, and the extraction was performed under 40 °C with 200 rpm shaking speed for 10 min. Methanol was used as the control solvent. (a) The content of the total flavonoids was determined by rutin method. (b) The content of isovitexin was determined by HPLC analysis. Data are the mean ± S.D. of three independent experiments.

HPLC analysis of isovitexin was performed on an Elite HPLC 1260 system (Agilent, USA) and a C18 column (4.6 × 250 mm, 5 μm, S. No. USNH017518, USA) with distilled water (A) and methanol (C), applying the following gradient program: 0–10 min C 20%; 10–20 min C 20%–55%; 20–30 min C 55% and 30–60 min C 100%. The flow ratio was 0.5 mL min^−1^ and the column temperature was 28 °C. The absorbance was detected at 320 nm. The chromatogram of the isovitexin is shown in Fig. 1. The retention time of isovitexin was 26.746 min (Fig. S4†). The content of isovitexin was calculated by a calibration curve established with the regression equation *y* = 22.556*x* − 175.65 (*R*^2^ = 0.997, Fig. S2†).

Flavonoids were detected using the rutin method.^25^ The sample was complemented by 80% (v/v) ethanol to 0.5 mL, then mixed with 30 μL NaNO_2_ solution (5.0%, w/w). After reacting for 6 min, 30 μL Al(NO_3_)_3_ solution (10%, w/w) was added to the mixture. It was shaken and reacted for 6 min to add the latter reaction reagents, which were 400 μL NaOH solution (4%, w/w) and 40 μL 80% (v/v) ethanol. The mixture was detected at 510 nm after 20 min. The content of flavonoids was calculated by establishing a rutin calibration curve. The regression equation was *y* = 0.1485*x* − 0.0081 (*R*^2^ = 0.9982, Fig. S3†).

### 2.5. Physicochemical characterization

In order to observe the micromorphology of the *Celtis sinensis* powder treated with different solvents, a field emission scanning electron microscope (FE-SEM, JSM7600F, JEOL Ltd.) was used.

### 2.6. Recovery of *Celtis sinensis* flavonoids from the DESs extraction solution

*Celtis* flavonoids were recovered from DES extraction solution by D-101 macroporous resin adsorption. 5.0 mL of DES extraction was put into a 10 mL flask and 2.0 g macroporous resin was added. Adsorption was operated at 25 °C and 150 rpm for 6 h. After filtration, the macroporous resin was desorbed with 5.0 mL 95% (v/v) ethanol solution at 25 °C and 150 rpm for 4 h. The *Celtis* flavonoids contents in the DES extraction solution *C*_0_, adsorption solution *C*_1_ and desorption solution *C*_2_ were determined. The adsorption rate of the macroporous resin and the desorption rate of 95% ethanol were calculated. The recovery process was repeated three times, and the averaged results were calculated. The adsorption rate *A* and resolution rate *B* were calculated according to the formula.*A* (%) = (*C*_0_ − *C*_1_)/*C*_0_ × 100%*A* – adsorption rate; *C*_0_ – concentration of total flavonoids in the extract, mg mL; *C*_1_ – concentration of total flavonoids in the adsorption solution, mg mL^−1^.*B* (%) = *C*_2_/(*C*_0_ − *C*_1_) × 100%*B* – resolution rate; *C*_2_ – concentration of the total flavonoids in the resolution solution, mg mL^−1^.

### 2.7. Animals

Female BALB/c mice aged 8 to 14 weeks were purchased from the Experimental Animal Center of Yangzhou city (Jiangsu, China). Animal welfare and experimental procedures were subjected to the Guide for the Care and Use of Laboratory Animals (National Institutes of Health, USA), and the study protocol was approved by the Animal Care and Protection Committee of Nanjing University-Gulou Hospital (SYXK 2004-0013). The authors confirmed that all animals received humane care, and all animal experiments were performed in accordance with the relevant guidelines and regulations. All of the authors complied with the ARRIVE guidelines experiments.

### 2.8. Cell viability by MTT assay

Cell viability was determined by MTT (4 mg mL^−1^) assay. T lymphocytes (7.5 × 10^5^/well) were inoculated in 96-well plates in 1640 medium. After incubating for 24 h, 20 μL of the MTT reagent was added, and each well was incubated for another 4 h. Then, 200 μL of dimethyl sulfoxide (DMSO) was added to dissolve the formazan crystals, and an absorbance at 450 nm was recorded by using a microplate reader to determine the cell viability. Each assay was performed in three replicates.

### 2.9. Mouse T lymphocyte proliferation *in vitro*

The mouse T lymphocytes were seeded and cultured in 96-well round-bottom plates at a density of 7.5 × 10^5^ cells, and stimulated with 5 μg mL^−1^ of concanavalin A (Con A) in the various concentrations of extracts (25–200 μg mL^−1^). After all groups were incubated for 48 h, each well was determined by the MTT assay. Each assay was performed in three replicates.

### 2.10. Cell apoptosis assay

Cells were measured by flow cytometry of the FITC-conjugated annexin V and PI. Annexin V+ cells were considered as apoptotic cells, while annexin V+/PI cells were considered as apoptotic cells in the early phase.

### 2.11. Western blot

Cells isolated from the lymph nodes were cultured in RPMI 1640 medium at a density of 1 × 10^7^ cells per well in a 6-well plate, and stimulated with Con A (5 μg mL^−1^). The lysed proteins were separated by SDS-PAGE and electrophoretically transferred to PVDF membranes (Millipore, Bedford, MA). After treatment with blocking buffer in 5% BSA at room temperature for 1 h, the membranes were incubated with primary antibodies at 4 °C overnight, and the secondary antibody was incubated at room temperature for 2 h. The protein content was detected by ECL luminescence method.

## 3. Results and discussion

### 3.1. Preparation of various types of DES

Several methods, including heating, evaporation, and freeze drying, can be used to prepare DES.^26^ In this work, the heating method was adopted because the heating method is simple. A number of reagents were tried as DES components, which are choline chloride, betaine, ethylene glycol, ethylamine hydrochloride, 1,2-propanediol, tetrapropylammonium bromide, glycerin, urea, lactic acid, malonic acid and glycolic acid.

Compared with traditional organic solvents, DES has higher viscosity and slower mass transfer, which is one of the main obstacles to the application of DES in the extraction field. Therefore, DES with lower viscosity and better fluidity should be selected when extracting. Adding water can not only reduce the viscosity of DES, but also change the polarity of DES. Combined with the research reports on the extraction of flavonoids^15–19,27^ in plants, hydrophilic DESs were selected to extract *Celtis sinensis*. In order to achieve the low price, easy preparation and environmental friendliness, choline chloride, betaine, ethylamine hydrochloride and tetrapylammonium bromide were preferentially selected as HBA, and some common acids, alcohols and sugars in the laboratory were selected as HBD. Based on the experiences of J. Cao *et al.*,^28^ 26 kinds of DESs were composed, and all of them were in the form of a clear liquid (Table 1).

### 3.2. Selection of DESs

The produced 26 kinds of DESs were used as solvents to extract flavonoids from *Celtis sinensis* leaves. At present, it has been reported that methanol is used as a solvent to extract flavonoids from *Celtis* leaves. It has also been reported that DES is usually very viscous, which impedes the mass transfer of compounds from the plant substrate to the extractive solvent.^11^ Therefore, in order to compare the extraction effect of methanol and reduce the viscosity of DES, the prepared DESs were mixed with deionized water at 7 : 3 (w/w) for screening. The results are shown in Fig. 1(a). It could be found that all of the DESs could extract the *Celtis* flavonoids with varied extraction yields, and the extraction yields of most DESs were significantly higher than that of methanol. The attained extraction yields of DES8, DES12 and DES17 were 80.85, 72.82 and 86.05 mg g^−1^, respectively, which were also the highest among the 26 DESs. Finally, DES8, DES12 and DES17 were selected for further design.

High-performance liquid chromatography (HPLC) analysis showed that *Celtis* contained anti-inflammatory flavonoids, including isovitexin, quercetin and luteolin. The content of isovitexin was the highest in the *Celtis* flavonoids. So, the 26 DESs were also used as solvents to extract isovitexin from *Celtis sinensis* leaves with the same conditions. The extraction rates of most DESs were higher than that of methanol, as shown in Fig. 1(b). DES23, DES24 and DES26, as the highest extractions among the 26 DESs, were selected to perform the next conditional optimization.

### 3.3. Tailoring the DES for higher extraction efficiency

#### 3.3.1. Optimization of the extraction conditions based on the extraction yield of the total flavonoids

**Fig. 2:**
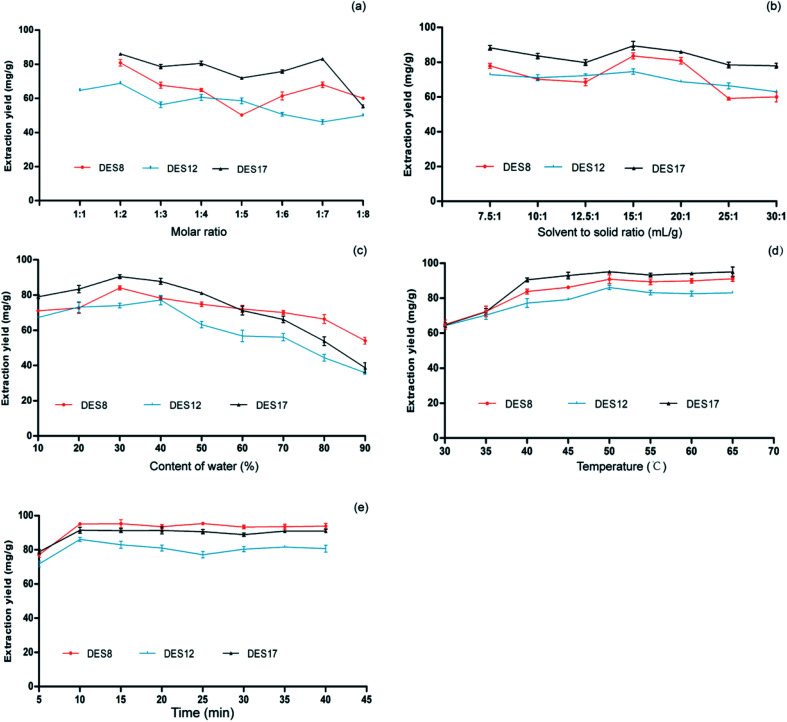
Optimization of the extraction conditions based on the extraction yield of *Celtis* flavonoids. (a) Effect of the molar ratio on the extraction yield of *Celtis* flavonoids. (b) Effect of the solvent-to-solid ratio on the extraction yield of *Celtis* flavonoids. (c) Effect of the content of water on the extraction yield of *Celtis* flavonoids. (d) Effect of temperature on the extraction yield of *Celtis* flavonoids. (e) Effect of time on the extraction yield of *Celtis* flavonoids. The extraction rates of the three DESs. Data are the mean ± S.D. of three independent experiments.

It is well known that the mole ratio of HBA to HBD is one of the important factors affecting the physico-chemical properties of a DES.^28^ Therefore, the effect of HBA-to-HBD molar ratios of DES8, DES12, and DES17 on the extraction yields of the total flavonoids was first investigated. DES8, DES12, and DES17 with the molar ratios ranging from 1 : 1 to 1 : 8 were prepared, and the extractions of total flavonoids were tested. The results are shown in Fig. 2(a). DES8 and DES17 could not form stable DESs when the molar ratio was 1 : 1, and the molar ratios of the three solvents showed a certain influence on the extraction yield. When the molar ratios of DES8 and DES12 decreased from 1 : 2 to 1 : 4, the amounts of HBDs increased, which led to the decrease in viscosity and surface tension of DES8 and DES12,^28^ thus improving their extraction efficiency. So, at the molar ratio of 1 : 2, DES8 and DES12 had the highest extraction rate. Comparatively, the extraction yield was the highest among the three DESs when the molar ratio of DES17 was 1 : 2. Through the above tailoring, the molar ratios of DES8, DES12 and DES17 were all chosen at 1 : 2 for further design. A low ratio of solvent to solid may lead to incomplete extraction, while a high ratio of solvent to solid may complicate the process and lead to DES waste. The effect of varied ratios between the DESs volume and *Celtis sinensis* leaves powder weight (7.5 : 1, 10 : 1, 12.5 : 1, 15 : 1, 20 : 1, 25 : 1, and 30 : 1) on the extraction rates of *Celtis* flavonoids among the three DESs were studied. As shown in Fig. 2(b), the extraction yields of the target *Celtis* flavonoids were higher when the ratios of solvent to solid were between 12.5 : 1 and 20 : 1. In this range, the mixture of *Celtis* leaves and DES may be thoroughly mixed. Considering the dosage and extraction yields of DESs, the DESs-to-solid ratio of 15 : 1 was used for the extraction of the target flavonoids.

The addition of water can reduce the viscosity of DES, which is conducive to the mass transport from plant matrices to solution. Furthermore, adding water to DES can modulate the polarity of DES, which may better match the polarity of the target compounds and improve the extraction yield. In order to determine the optimum water content in three kinds of DESs for *Celtis* flavonoids extraction, the extraction procedures were performed in DES8, DES12 and DES17 with different water contents (10–90%, w/w). The results are shown in Fig. 2(c). It could be observed that the extraction rates of the three DESs reached a maximum value when the water content was 30–50% (w/w). When the water content increased from 10 to 30% (w/w), the extraction yields obviously increased. However, the higher concentration of water in the three DESs resulted in a decrease of the target flavonoids extraction. The addition of water can effectively reduce the viscosity and has a good effect on the polarity of solvent, while the excessive water content could reduce the interaction between the DESs and flavonoids, which is not conducive to the increase of the polarity of the solvent mixture. The concentrations of 30% (w/w) water in DES8 and DES17 and 40% (w/w) water in DES12 were selected for subsequent experiments.

The *Celtis* flavonoid glycosides are adsorbed on plant substrates by physical adsorption and/or chemical interactions. Increasing the temperature is one of the most convenient methods to reduce the adsorption and/or interaction for desorption and dissolution of flavonoids on extraction solvents. Also, with the increase of temperature, the DESs viscosity will decrease and its diffusivity will increase, which promoted the release of *Celtis* flavonoids from plant substrate to DESs. The effect of temperature on the extraction rate of flavonoids from *Celtis* was investigated at 30, 35, 40, 45, 50, 55, 60, 65 and 70 °C. The results are shown in Fig. 2(d). The results showed that the extraction rates increased with the increase of temperature from 30 °C, and reached the maximum at about 50 °C for the three DESs. Therefore, the final temperature of 50 °C was selected for the time optimization. It was very important to select the appropriate extraction time to ensure the extraction balance of target *Celtis* flavonoids between the *Celtis sinensis* leaves powder and DESs–water mixture. The extraction time was between 5 and 40 min. The results are shown in Fig. 2(e). For all three DESs, the extraction rates and extraction time of *Celtis* flavonoids increased slowly in the first 10 minutes. After 15 minutes, the extraction time had little effect on the extraction rates, indicating that *Celtis* flavonoids reached their extraction equilibrium at around 10 min. Allowing for the improvement of the extraction rate and the shorter extraction time, 10 min was selected as the appropriate extraction time.

#### 3.3.2. Optimization of extraction conditions based on the extraction yield of isovitexin

**Fig. 3:**
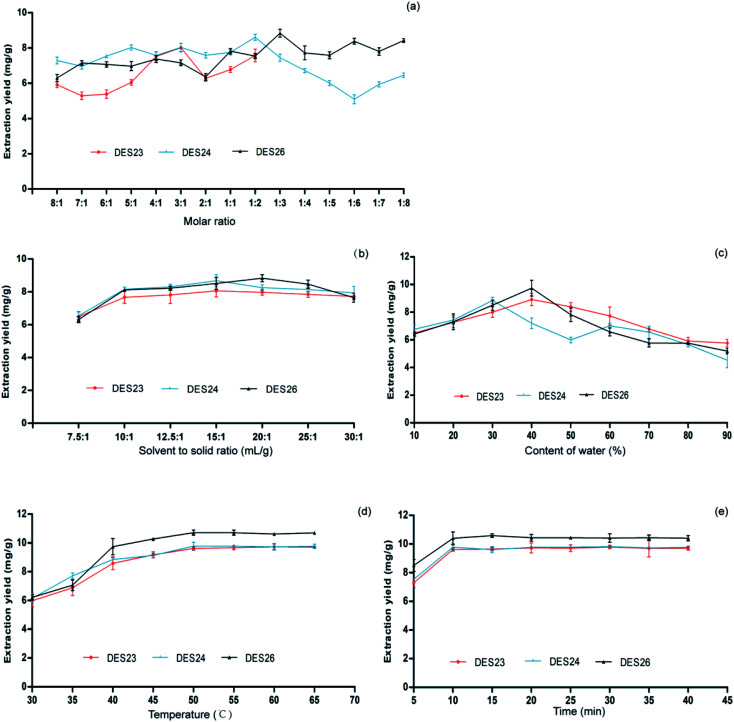
Optimization of the extraction conditions based on the extraction yield of isovitexin from *Celtis sinensis* leaves. (a) Effect of the molar ratio on the extraction yield of isovitexin. (b) Effect of the solvent-to-solid ratio on the extraction yield of isovitexin. (c) Effect of the content of water on the extraction yield of isovitexin. (d) Effect of temperature on the extraction yield of isovitexin. (e) Effect of time on the extraction yield of isovitexin. Data are the mean ± S.D. of three independent experiments.

DES is a low eutectic mixture generally composed of a hydrogen bond acceptor (HBA) and hydrogen bond donor (HBD) with a certain molar ratio, so the molar ratio was based on HBA and HBD from 1 : 1–1 : 8 in the extract of the total flavonoids. However, different kinds of sugars, organic acids and alcohol mixtures can also form liquids without distinguishing between the HBA and HBD,^29^ so a molar ratio range from 8 : 1 to 1 : 8 was used. Through preliminary screening, three DESs were selected for further tailoring by changing the mole ratio between component 1 and component 2, which were DES23, DES24, and DES26. The molar ratio ranging from 8 : 1 to 1 : 8 was prepared, and the extraction of isovitexin was tested. As shown in Fig. 3(a), DES23 could not form a stable DES at the molar ratios between 1 : 3 to 1 : 8. The extraction rates of isovitexin varied significantly, along with the change of the molar ratio between component 1 and component 2 in the three DESs. As a result, DES23 (3 : 1), DES24 (1 : 2), and DES26 (1 : 3) were chosen for the next tailoring.

The extraction rates of isovitexin extracted by three DESs increased with the increase of the DESs content when the *Celtis sinensis* leaves were mixed evenly. The results are show in Fig. 3(b). The extraction rates of isovitexin extracted by DES23 and DES24 from *Celtis sinensis* increased slightly with the increase of the DES volume before the DESs solvent-to-solid ratio reached 15 : 1, and then remained unchanged with the further increase of the DES volume. In contrast, the extraction rates of isovitexin extracted by DES26 increased with the increase of the DESs volume before the DESs solvent-to-solid ratio reached 20 : 1. So, a solvent-to-solid ratio of 15 : 1 was used in DES23 and DES24 for the extraction of isovitexin, and a solvent-to-solid ratio of 20 : 1 was used in DES26 for the extraction of isovitexin.

It could effectively adjust the polarity, reduce the viscosity and increase the mass transfer by adding a certain amount of water into DESs during the extraction process.^30^ The results are shown in Fig. 3(c). For the three DESs, the extraction rates showed the same trend of increasing first and then decreasing. When the water content was less than 40% (w/w), the extraction rates of isovitexin extracted by DES23 and DES26 increased with the increase of the water content. Furthermore, when the water content was less than 30% (w/w), the extraction rate of isovitexin extracted by DES24 increased with the increase of the water content.^31^ On the one hand, the viscosity of DES decreased with the increase of the water content. Therefore, the mass transfer between DES and *Celtis sinensis* leaves powder would increase, which led to the increase of the extraction rate. On the other hand, adding water would change the polarity of DESs. When the water content was more than 30% (w/w) in DES24 or more than 40% (w/w) in DES23 and DES26, the extraction efficiency decreased with the further increase of water content. This phenomenon could be explained by the hydrogen bond weakening between the components of DESs due to the hydrogen bond strengthening between water and DES.^32^ In other words, the hydrogen bond network of DES was destroyed by adding too much water. Accordingly, DES24 with 30% water content (w/w) and DES23 and DES26 with 40% (w/w) water content were selected for the next optimization of the extraction conditions.

The extraction efficiency is often affected by diffusion, viscosity and solubility, which are influenced by temperature.^33^ At higher temperature, the viscosity of the solvent decreases, and the diffusivity and solubility increase, which were conducive to the penetration of target compounds from plant material into solvent. The effect of temperature on the extraction rates of isovitexin extracted by three DESs was investigated from 30 °C to 70 °C. The results are shown in Fig. 3(d). The extraction rates of isovitexin extracted by three DESs increased obviously with the increase of the extraction temperature from 30 °C to 50 °C, reached the maximum value at 50 °C, and then remained unchanged. The extraction rates of isovitexin decreased slightly when the temperature was higher than 55 °C, which might result from the decomposition of isovitexin at high temperature. Thus, 50 °C was chosen as the optimal extraction temperature.

The extension of the extraction time has a positive effect on the total extraction rate. However, too long of an extraction time will waste energy. It is necessary to find a suitable extraction time. The extraction time was varied from 5 min to 45 min, and the results are shown in Fig. 3(e). The extraction rates of isovitexin extracted by the three DESs increased with the extension of time, and tended to be stable after 10 min. So, the time of 10 min was selected as the appropriate extraction time.

**Fig. 4:**
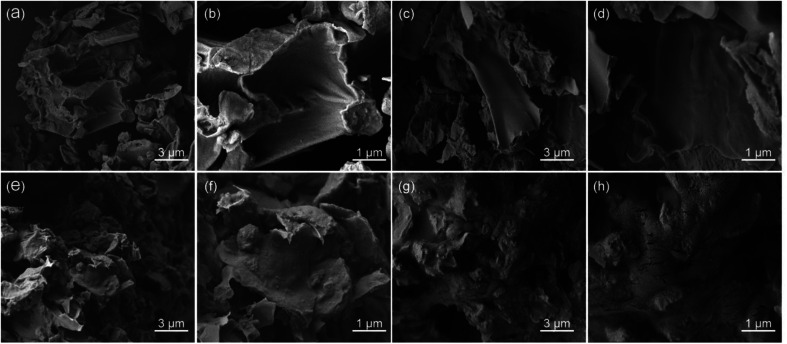
SEM images of the *Celtis sinensis* samples: (a and b) raw materials, (c and d) treated with water, (e and f) treated with methanol, (g and h) treated with 30 wt% aqueous solution of DES17.

In order to investigate the extraction mechanism, the morphology of *Celtis sinensis* was observed by SEM. The flavonoid extraction yield of DES17 was the highest among the 26 DESs at the initial screening (Fig. 1(a)), so DES17 was chosen for comparison with the raw *Celtis* sample, the sample treated with water, and methanol. As shown in Fig. 4(a and b), the surface tissue of the raw *Celtis* sample was mostly smooth, and the roughness of some areas can be ignored. In contrast, the samples treated with water and methanol gradually showed obvious physical changes, such as the increase of surface bulges and folds (Fig. 4(c–f)). In the sample treated with 30 wt% aqueous solution of DES17, the surface structure was significantly damaged, generating many cracks (Fig. 4(g and h)). This may be because DES can dissolve and/or hydrolyze cellulose, leading to cell wall destruction. Therefore, a higher number of target compounds were exposed to the extract.

### 3.4. Recovery of *Celtis* flavonoids from the DESs extraction solution

**Fig. 5:**
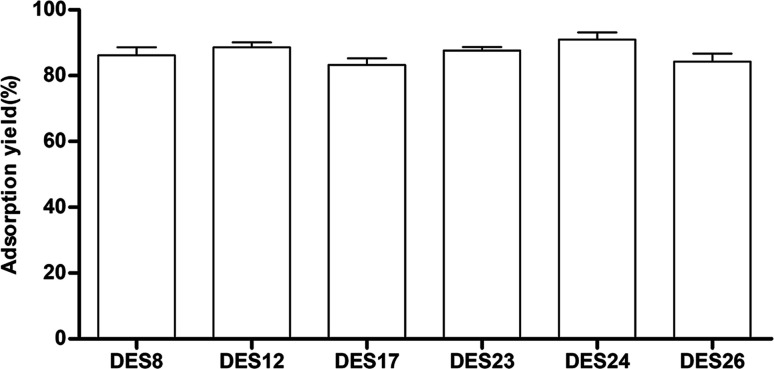
Recovery of *Celtis sinensis* flavonoids from the DES extraction solution with D-101 macroporous resin purification.

Because of the negligible vapor pressure and high water miscibility of the six DESs, it was a challenging task to recover the target compounds from the DESs extraction solution. Several methods for the recovery of the target compounds were proposed, including the application of antisolvents, recrystallization, back extraction, chromatographic techniques, and countercurrent separation.^34,35^ In this work, D-101 macroporous resin was used to extract the target *Celtis* flavonoids from the DESs extraction. The results are show in Fig. 5. The results showed that D-101 macroporous resins could adsorb the *Celtis* flavonoids extracted by different DESs with diverse adsorption yields, and the adsorption yields were all more than 85%. The D-101 resin adsorbing *Celtis* flavonoids were washed twice with 95% (v/v) ethanol solution, and almost all of the *Celtis* flavonoids could be freed by the ethanol solution. Therefore, the D-101 resin was effective for the recovery of *Celtis* flavonoids.

### 3.5. Anti-inflammatory activity of the *Celtis sinensis* extracted by different solvents

**Fig. 6:**
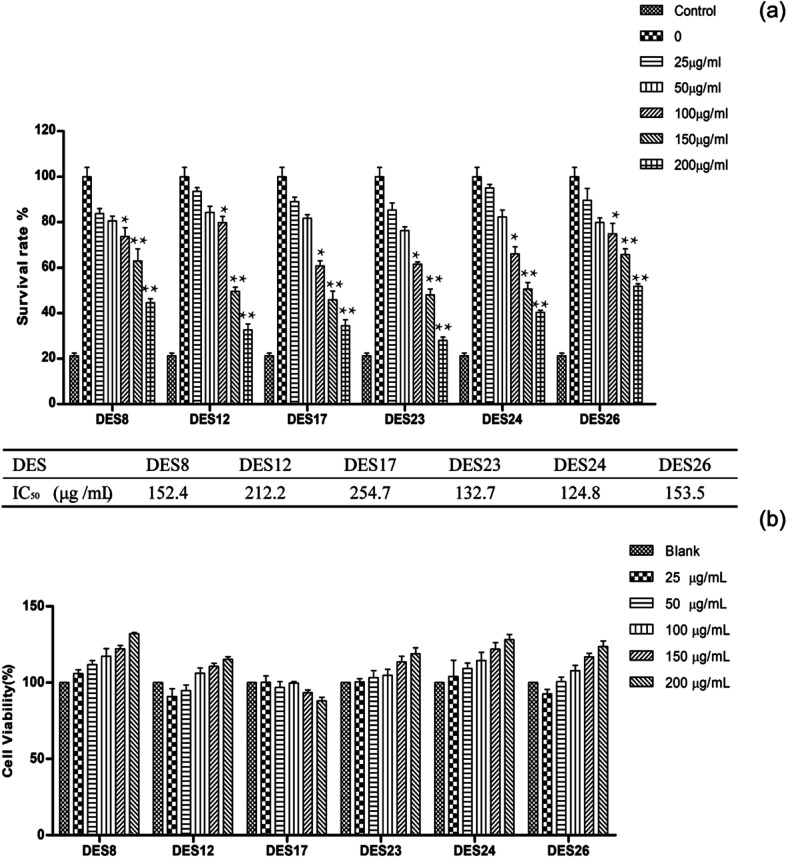
Anti-inflammatory activity of the DESs extracts of *Celtis sinensis*. T lymphocytes were seeded in 96-well plates, and incubated with or without Con A (2.5 μg mL^−1^) at various concentrations (25, 50, 100, 150, 200 μg mL^−1^) of *Celtis sinensis* extracts. (a) T lymphocytes were incubated with Con A and *Celtis sinensis* extracts, and the proliferation was determined by MTT assay after 48 h. (b) T lymphocytes were incubated with *Celtis sinensis* extracts, and the cytotoxicity of the *Celtis sinensis* extracts on the T lymphocytes was determined by MTT assay after 24 h. Data are the mean ± S.D. of three independent experiments. *P < 0.05, **P < 0.01 *vs.* model group (Con A-activated T cells).

After the extracts were purified by resin, the final crude extracts for exploring active experiments were obtained. Studies have shown that the main pharmacological effect of the *Celtis sinensis* leaves extract include analgesia, antiasthmatic, anti-inflammatory, and the leaves of *Celtis sinensis* were used as folk medicine to treat dermatitis caused by urushiol. Among those, we study the attenuating effect of crude extracts isolated from DES on inflammation. Con A-activated T lymphocyte was used to explore the mechanism of the extracts. Con A is a plant lectin, which can induce the mitotic activity of T lymphocytes and increase the production of inflammatory cytokines, for instance, IL-2, TNF-α and IFN-γ.^6^ As shown in Fig. 6(a), the inhibition rates increased along with the increase of concentrations. The data suggested that the anti-inflammatory activity was more effective when increasing the concentration. The inhibition rate was over 70% when the concentration reached 200 μg mL^−1^ of DES24 crude extract, and the value of IC_50_ was 124.8 μg mL^−1^. To prove the anti-inflammatory activity of the extractions, a MTT assay was used to determine the growth inhibition effect of the crude extracts on T lymphocyte. In Fig. 6(b), the results show that none of the six extracts showed significant toxicity to normal T-lymphocyte. The data indicated that the six extractions had good anti-inflammatory effects, and the extractions of DES24 had the best anti-inflammatory activity.

High-performance liquid chromatography (HPLC) analysis showed that *Celtis* contained anti-inflammatory flavonoids, including isovitexin, quercetin and luteolin.^36,37^ The crude extractions contained an abundance of *Celtis* flavonoids in previous experiments. Furthermore, isovitexin is the most abundant flavonoid in the crude extractions. It has anti-inflammatory pharmacological properties.^38^ Therefore, the crude extraction obtained has an effective anti-inflammatory activity.

### 3.6. The protein expression level of P-AKT, cleaved-PARP and BCL-2

**Fig. 7:**
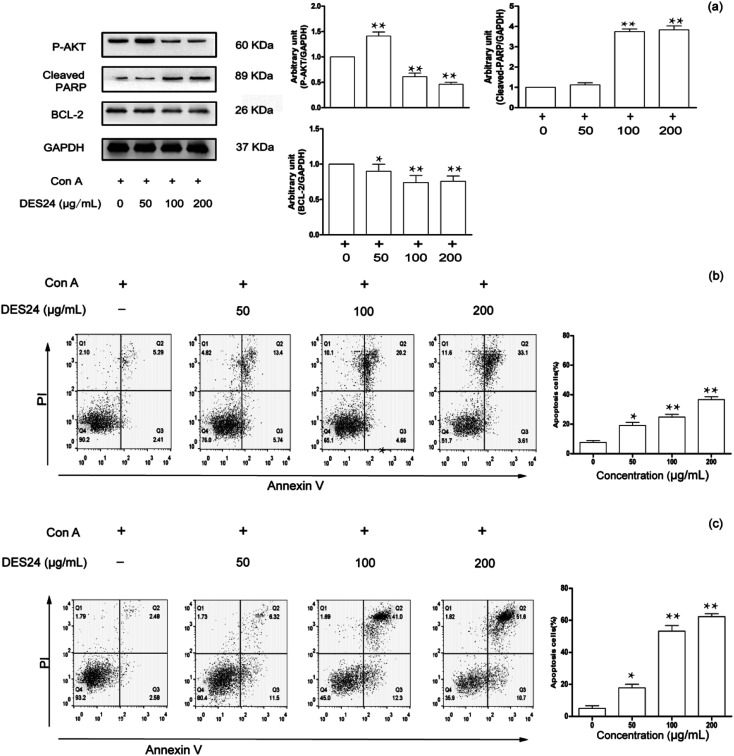
Effect of the DES extract of *Celtis sinensis* (DES24) on the apoptosis of Con A-activated T cells. (a) Con A-activated T cells were incubated with different concentrations of DES24 for 48 h, and the expression levels of P-AKT, cleaved-PARP and BCL-2 were evaluated by WB, and the relative band density was analyzed using Image J. Con A-activated T cells were incubated with different concentrations (50, 100, 200 μg mL^−1^) of DES24 for 24 h (b) or 48 h (c), and cells were harvested for annexin V/PI double staining. The apoptosis induced by DES24 was determined by flow cytometric analysis. *P < 0.05, **P < 0.01 *vs.* control (Con A-activated T cells).

To further research the underlying mechanisms of the extractions on T lymphocyte apoptosis and signaling pathways, the purified crude extraction extracted by DES24 was selected as a research object to verify the result in Fig. 5. We further examined the effect of crude extraction on P-AKT, cleaved-PARP and BCL-2 in activated T lymphocyte. These results are shown in Fig. 7(a). In this study, the results showed that cleaved-PARP was significantly enhanced in a dose-dependent manner, and P-AKT and BCL-2 decreased in turn as the concentrations increased. Hence, the crude extraction dampens pro-inflammatory signaling, and promotes the resolution of inflammation.

### 3.7. Features of apoptotic T lymphocyte measured by flow cytometry

In most cases, apoptosis was selected at a specific stage of the cell cycle, and necrosis was induced by excessively high concentrations of the drugs. Annexin V combined with PI has been proved to be an excellent probe for distinguishing living cells, necrotic cells, early cells and late apoptotic cells. The inhibitory effect of DES24 extraction was demonstrated on activated T lymphocyte by flow cytometry. The results are shown in Fig. 7. Three kinds of concentration increases in the gradient were selected. The cell apoptosis increased with the increase of the drug concentration. Cell apoptosis was 36.7% when fostering the activated T lymphocyte for 24 h. It meant that the extraction had a certain inhibitory rate, but was not satisfactory. Hence, we changed the time from 24 h to 48 h for comparison. In Fig. 7(c), at the concentration of 200 μg mL^−1^, the cell apoptosis rose from 36.7% to 62.3% when the time was changed from 24 h to 48 h.

## 4. Conclusions

In this work, it has been demonstrated that deep eutectic solvents can be used as alternative solvents for efficient extraction of flavonoids from *Celtis sinensis* leaves. Experimental design approaches were employed to obtain the optimal conditions based on the extraction yield of total flavonoids and isovitexin. Comparing with methanol, DESs showed advantages in non-flammability, non-volatility, high extraction yield and short extraction time. Meanwhile, it was proved that D-101 macroporous resin was an effective method for enrichment and purification of *Celtis* flavonoids. Among the selected extractions, *Celtis* flavonoid extracts by DES24 exhibited relatively higher anti-inflammatory activity against T cells through inducing apoptosis of inflammatory T cells. The developed method allows for the extraction of flavonoids from *Celtis sinensis* with excellent accuracy and repeatability. Therefore, the DES technique was a green and integrated strategy for the extraction of bioactive ingredients from plant materials.

## Conflicts of interest

The authors declare that they have no known competing financial interests or personal relationships that could have appeared to influence the work reported in this paper.

## Supplementary Material

RA-011-D1RA01848E-s001

## References

[cit1] Adedapo A. A., Jimoh F. O., Afolayan A. J., Masika P. J. (2009). Rec. Nat. Prod..

[cit2] El-Alfy T. S., El-Gohary H. M. A., Sokkar N. M., Hosny M., Al-Mahdy D. A. (2011). Sci. Pharm..

[cit3] Akin D., Durak Y., Uysal A., Gunes E., Aladag M. O. (2016). Drug Chem. Toxicol..

[cit4] Martins J. L. R., Rodrigues O. R. L., Da Silva D. M., Galdino P. M., de Paula J. R., Rom O W., Da Costa H. B., Vaz B. G., Ghedini P. C., Costa E. A. (2014). J. Ethnopharmacol..

[cit5] Abbott A. P., Capper G., Davies D. L., Rasheed R. K., Tambyrajah V. (2003). Chem. Commun..

[cit6] Dai Y., Van Spronsen J., Witkamp G. J., Verpoorte R., Choi Y. H. (2013). J. Nat. Prod..

[cit7] Rojas O. G., Nakayama T., Hall S. R. (2021). Ceram. Int..

[cit8] Shishov A., Bulatov A., Locatelli M., Carradori S., Andruch V. (2017). Microchem. J..

[cit9] Nemati M., Farajzadeh M. A., Mohebbi A., Sehatkhah M. R., Mogaddam M. R. A. (2020). Microchem. J..

[cit10] Paiva A., Craveiro R., Aroso I., Martins M., Reis R. L., Duarte A. R. C. (2014). ChemInform.

[cit11] Nam M. W., Zhao J., Lee M. S., Jeong J. H., Lee J. (2015). Green Chem..

[cit12] Dai Y., Witkamp G. J., Verpoorte R., Choi Y. H. (2013). Anal. Chem..

[cit13] Gu T., Zhang M., Tan T., Chen J., Li Z., Zhang Q., Qiu H. (2014). Chem. Commun..

[cit14] Gao M. Z., Cui Q., Wang L. T., Meng Y., Fu Y. J. (2020). Microchem. J..

[cit15] Jeong K. M., Zhao J., Jin Y., Heo S. R., Lee J. (2015). Arch. Pharmacal Res..

[cit16] Lee J., Jeong K. M., Lee M. S., Nam M. W., Zhao J., Jin Y., Lee D.-K., Kwon S . W., Jeong J. H. (2015). J. Chromatogr. A.

[cit17] Moghaddam M., Miran S. N. K., Pirbalouti A. G., Mehdizadeh L., Ghaderi Y. (2015). Ind. Crops Prod..

[cit18] Choi Y. H., Oomen W. W., Begines P., Mustafa N. R., Wilson E. G. (2020). Molecules.

[cit19] Bi W., Tian M., Row K. H. (2013). J. Chromatogr. A.

[cit20] Wei Z. F., Wang X. Q., Peng X., Wang W., Zhao C. J., Zu Y. G., Fu Y. J. (2015). Ind. Crops Prod..

[cit21] Mei N., Guo X., Ren Z., Kobayashi D., Wada K., Guo L. (2017). J. Environ. Sci. Health, Part C: Environ. Carcinog. Ecotoxicol. Rev..

[cit22] Hotta E., Tamagawa-Mineoka R., Katoh N. (2013). Eur. J. Dermatol..

[cit23] Ude C., Schubert-Zsilavecz M., Wurglics M. (2013). Clin. Pharmacokinet..

[cit24] Durant-Archibold A. A., Santana A. I., Gupta M. P. (2018). J. Ethnopharmacol..

[cit25] Abbott A. P., Boothby D., Capper G., Davies D. L., Rasheed R. K. (2004). J. Am. Chem. Soc..

[cit26] Volikakis G. J., Efstathiou C. E. (2000). Talanta.

[cit27] Delazar A., Lasheni S., Fathiazad F., Nahar L. (2010). Rec. Nat. Prod..

[cit28] Lu C., Cao J., Wang N., Su E. (2016). Med. Chem. Commun..

[cit29] Liu L., Li S., Chen Z. (2012). J. Pharm. Biomed. Anal..

[cit30] Li J., Han Z., Zou Y. (2015). RSC Adv..

[cit31] Naser J., Mjalli F., Jibril B., Al-Hatmi S., Gano Z. (2013). Int. J. Chem. Eng. Appl..

[cit32] Shah D., Mjalli F. S. (2014). Phys. Chem. Chem. Phys..

[cit33] Nam M., Zhao J., Jeong J., Lee J. (2015). Green Chem..

[cit34] Passos H., Tavares D. J. P., Ferreira A. M., Freire M. G., Coutinho J. A. P. (2016). ACS Sustainable Chem. Eng..

[cit35] Wang Y., Hou Y., Wu W., Liu D., Ji Y., Ren S. (2016). Green Chem..

[cit36] Liu Y., Garzon J., Friesen J. B., Zhang Y., Mcalpine J. B., Lankin D. C., Chen S. N., Pauli G. F. (2016). Fitoterapia.

[cit37] Shinohara Y., Tsukimoto M. (2018). Front. Pharmacol..

[cit38] Gyung K. H., Subin C., Jongsung L., Han H. Y., Deok J., Keejung Y., Hyo Y. D., Gi-Ho S., Seungihm L., Suntaek H. (2018). Evid. Based Complement. Alternat. Med..

