# Supplementary material for: Efficient extraction of bioactive flavonoids from Celtis sinensis leaves using deep eutectic solvent as green media
Source: RSC Adv. 2021 May 18;11(29):17924–35. doi: 10.1039/d1ra01848e (PMC9033205; doi:10.1039/d1ra01848e)
Supplement: RA-011-D1RA01848E-s001 [file RA-011-D1RA01848E-s001.pdf]

Electronic Supplementary Material (ESI) for RSC Advances.
This journal is © The Royal Society of Chemistry 2021

Supplementary Material

Efficient extraction of bioactive flavonoids from *Celtis sinensis* leaves using deep eutectic solvent as green media

Lei Wang[a,c,1], Xianying Fang[a,c,1], Yang Hu[a], Yiwei Zhang[a], Zhipeng Qi[a], Jie Li[a], Linguo Zhao[a,b,c *]

[a] *College of Chemical Engineering, Nanjing Forestry University, Nanjing 210037, China;*

[b] *Co-Innovation Center for Sustainable Forestry in Southern China, Nanjing Forestry University, Nanjing 210037, China;*

[c] *Jiangsu Co-Innovation Center of Efficient Processing and Utilization of Forest Resources, Nanjing Forestry University, Nanjing 210037, China.*

[1] *These authors contributed equally to this work*

***Corresponding author:** Linguo Zhao [*]

Tel.: +86-25-85427396

E-mail address: njfu2304@163.com

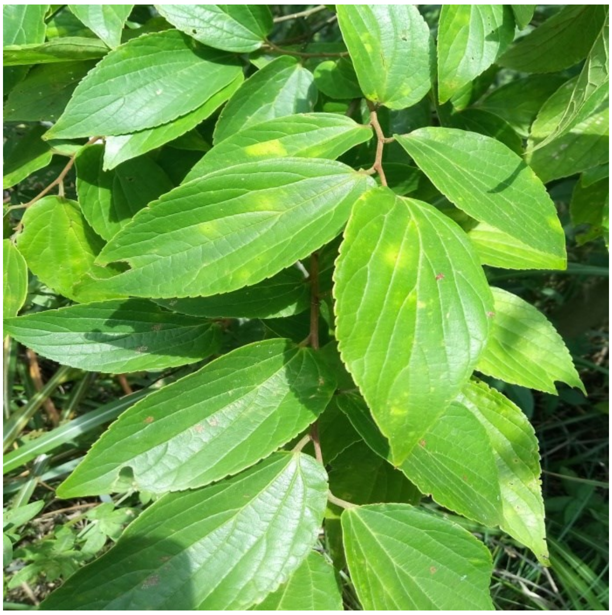

Fig. S1 Schematic representation of *Celtis sinensis* leaves.

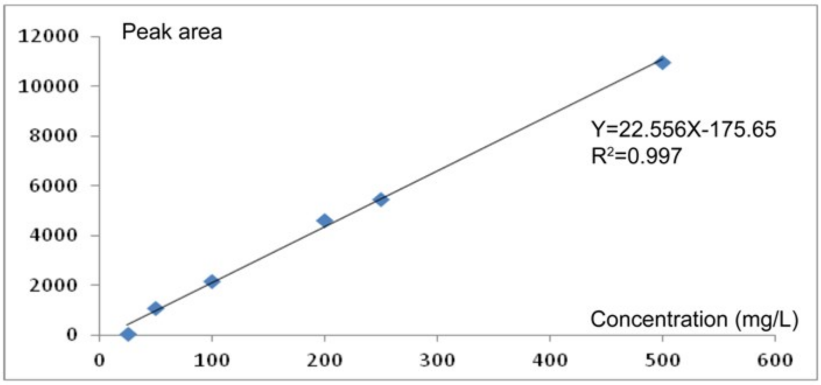

Fig. S2 Content of isovitexin was calculated by a calibration curve established with the regression equation by HPLC. (X is concentration (mg/L) of isovitexin, Y is the peak area of isovitexin)

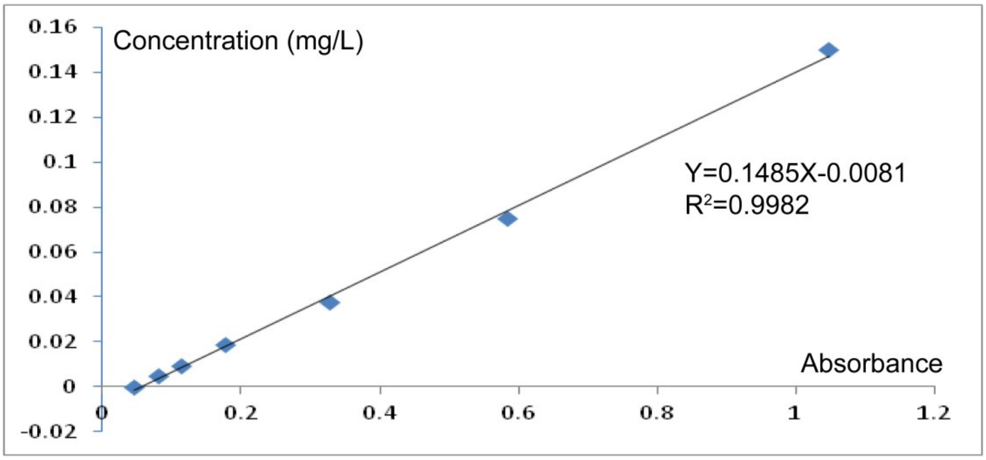

Fig. S3 Rutin was used as the standard substance to establish curve to measure total flavonoids. (X is absorbance of rutin, Y is the concentration (mg/mL) of rutin)

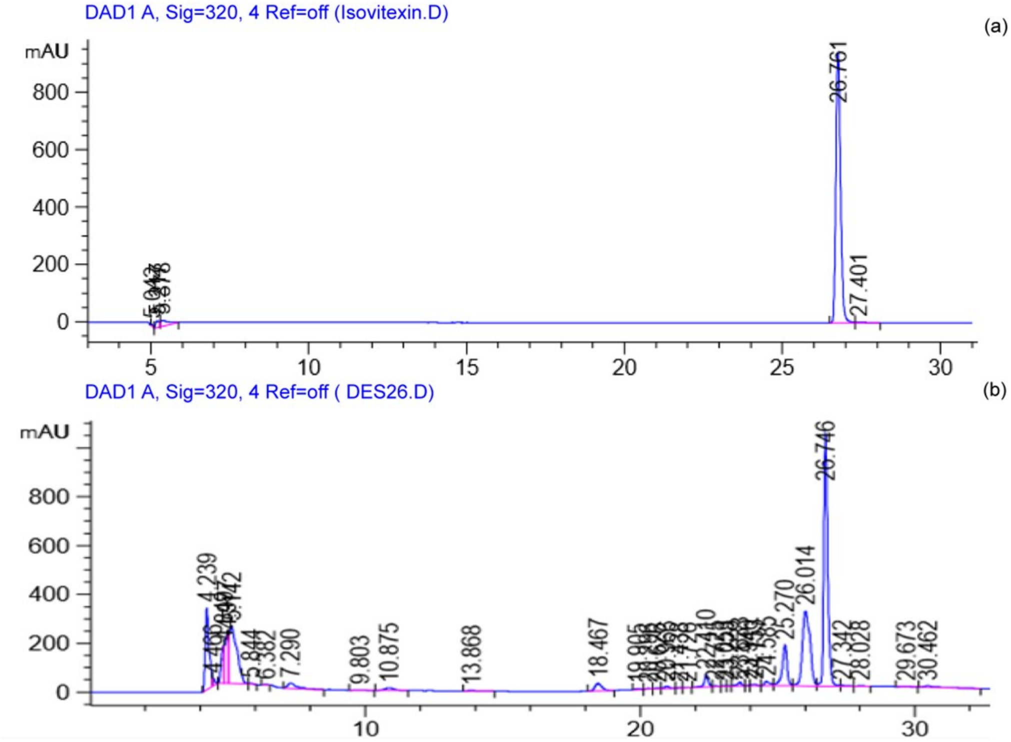

Fig. S4 HPLC-chromatogram for analysis of isovitexin extracted from *Celtis sinensis*. (a) HPLC-chromatogram for analysis of isovitexin. (b) HPLC-chromatogram of DES26 extract.
